# Comparison of adenoma detection rate using the novel 5-LED vs xenon-light endoscopic system: Propensity score matching analysis

**DOI:** 10.1055/a-2760-6529

**Published:** 2025-12-19

**Authors:** Tatsuhiro Ito, Satoshi Osawa, Takanori Yamada, Keisuke Inagaki, Tomohiro Takebe, Satoru Takahashi, Shunya Onoue, Kiichi Sugiura, Natsuki Ishida, Tomoharu Matsuura, Mihoko Yamade, Moriya Iwaizumi, Yasushi Hamaya, Ken Sugimoto

**Affiliations:** 1 12793 First Department of Medicine, Hamamatsu University School of Medicine, Hamamatsu, Japan; 2 12793 Department of Advanced Medical Science for Regional Collaboration, Hamamatsu University School of Medicine, Hamamatsu, Japan; 3 12793 Department of Endoscopic and Photodynamic Medicine, Hamamatsu University School of Medicine, Hamamatsu, Japan; 4 First Department of Medicine, Hamamatsu University School of Medicine, Hamamatsu, Japan; 5 12793 Department of Laboratory Medicine, Hamamatsu University School of Medicine, Hamamatsu, Japan

**Keywords:** Endoscopy Lower GI Tract, Polyps / adenomas / ..., Diagnosis and imaging (inc chromoendoscopy, NBI, iSCAN, FICE, CLE...), Endoscopic resection (polypectomy, ESD, EMR, ...)

## Abstract

**Background and study aims:**

Olympus’s new endoscopic system, EVIS X1, features five-LED illumination and a novel complementary metal-oxide-semiconductor (CMOS) image sensor distinct from conventional charge-coupled devices (CCDs), potentially improving colorectal adenoma detection rates (ADRs). This study compared ADR and related indicators between the EVIS X1 system and the conventional EVIS LUCERA ELITE, a xenon-light system.

**Patients and methods:**

Of 4,915 colonoscopies performed between September 2020 and April 2023, 814 EVIS X1 and 953 LUCERA cases met inclusion criteria. After propensity score matching to balance baseline characteristics, 660 patients per group were analyzed. Outcomes included ADR, polyp detection rate (PDR), adenomas per colonoscopy (APC), and polyps per colonoscopy (PPC). Subgroup analysis assessed the impact of CMOS-equipped scopes within the X1 group.

**Results:**

ADR was slightly higher in the X1 group (36.1%) than the LUCERA group (32.1%), although not statistically significant ( *P* = 0.147). APC (0.77 vs. 0.61, *P* = 0.034) and PPC (0.95 vs. 0.75, *P* = 0.023) were significantly higher with X1. Within the X1 group, scopes with CMOS sensors achieved a significantly higher ADR (41.9%) compared with those without. Mean size of polyps detected was smaller with CMOS than with CCD scopes. Multivariate analysis identified age > 60 years, male sex, positive fecal occult blood test, and use of the X1 system with CMOS scopes as independent predictors of higher ADR.

**Conclusions:**

The EVIS X1 system may have the potential to improve adenoma detection, particularly when used with CMOS sensor-equipped scopes. These findings suggest potential benefits for colorectal cancer screening, although further large-scale studies are warranted for validation.

## Introduction

Colonoscopy is a reliable method for detecting and diagnosing colorectal neoplasms and polypectomy has been shown to be both diagnostically and therapeutically effective in reducing incidence of colorectal cancer 1 . However, it has been reported that conventional endoscopic systems using white light imaging (WLI) with a xenon light source miss approximately 25% of colonic lesions 2 3 . Randomized studies have investigated several factors contributing to these differences. Lesions may be overlooked due to poor bowel preparation 4 , haustral folds, technical factors 5 , and lesion-related characteristics such as flat and depressed morphologies that are difficult to detect 3 . Therefore, improvements in colonoscopy procedures and various colonoscopy modalities are considered to overcome these limitations.

Recent advances in endoscopic technology have improved accuracy of endoscopy using image-enhanced endoscopy (IEE) for lesions that are difficult to observe using conventional WLI. IEE modalities such as narrow-band imaging (NBI) 6 , blue laser imaging/blue light imaging (BLI) 7 , linked color imaging (LCI) 8 , and i-scan 9 , which can be switched to conventional WLI by pressing a button on the endoscope, are now available. Recent reports have shown that use of these IEEs increases the detection rate for colorectal adenomas, in addition to their role in differential diagnosis of non-neoplastic and neoplastic lesions 6 10 . However, screening the entire colon of every patient twice using both WLI and IEE is not widely practiced due to time constraints and the complexity of the procedure, despite evidence suggesting that it may be more beneficial than WLI alone. In general clinical practice, IEE is typically used only after a suspicious lesion has been detected by WLI, primarily for differential diagnosis. Consequently, WLI remains the gold standard for lesion detection in screening colonoscopies. To date, many studies have compared high-definition white light endoscopy (HD-WLE) with standard-definition WLE (SD-WLE) in xenon light source endoscope systems 11 , and meta-analyses have shown that HD-WLE has a certain level of significance in detecting colon polyps 12 . The European Society of Gastrointestinal Endoscopy, therefore, recommends use of high-resolution endoscopes in clinical practice if possible 13 . However, the advantage of HD-WLE remains limited, and it is desirable that examinations using higher-resolution WLI than ever before could improve lesion detection.

**Fig. 1:**
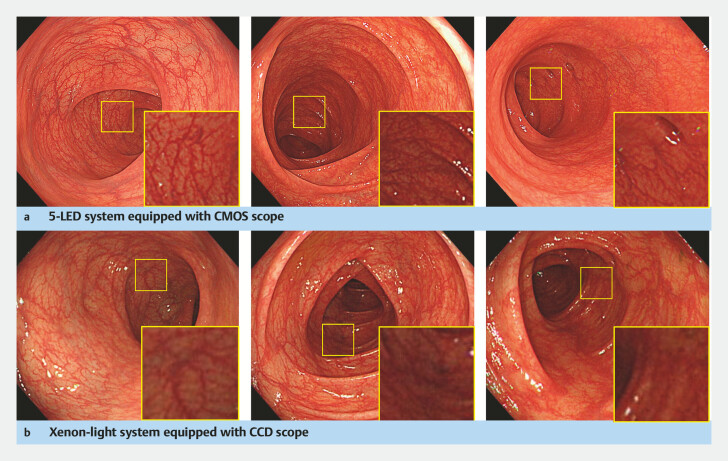
Typical endoscopic images obtained with a five-LED system and a conventional xenon light system. The squares indicate magnified views of a distant area, located 10 to 15 cm from the tip, highlighting differences in light intensity and visibility. **a** Five-LED system equipped with a CMOS scope: EVIS X1 with CF-XZ1200I. **b** Xenon light system equipped with a CCD scope: EVIS LUCERA ELITE with CF-HQ290ZI.

The new EVIS X1 colonoscopy system (Olympus Medical Systems, Tokyo, Japan) features novel five-LED illumination technology and is compatible with a novel complementary metal-oxide-semiconductor (CMOS) image sensor distinct from a conventional charge-coupled device (CCD). These new technologies provide high-resolution WLI not available with traditional xenon light endoscopy systems ( Fig. 1 ). However, the magnitude of the additional effect of detecting colorectal lesions in clinical practice remains unclear. In this study, we investigated the impact of the X1 system on colorectal polyp detection compared with the EVIS LUCERA ELITE system, a xenon light system, in a general clinical setting. The primary objective of this study was to compare adenoma detection rate (ADR) and polyp detection rate (PDR) before and after implementation of high-definition colonoscopy. In this study, in addition to differences in the endoscope system depending on the light source, we also examined detection of lesions depending on the type of scope equipped with the CMOS image sensor.

## Patients and methods

### Study design and patients

This retrospective cohort study was conducted at Hamamatsu University School of Medicine. The study was approved by the institutional review board (IRB approval number: 24–012) and was conducted in accordance with the Japanese Ethical Guidelines for Medical and Health Research Involving Human Subjects.

We reviewed medical records of patients who underwent colonoscopy between September 2020 and April 2023. Endoscopic findings and pathological diagnosis results were extracted using the Solemio QUEV system (Olympus Medical Systems) and the hospital electronic medical record system. A total of 4,915 consecutive colonoscopies performed at Hamamatsu University Hospital were identified. Inclusion criteria were colonoscopies performed using either the EVIS X1 or EVIS LUCERA ELITE systems. Exclusion criteria were as follows: emergency endoscopy; patients presenting with abdominal symptoms, hematochezia/melena; a history of inflammatory bowel disease or polyposis; prior colon resection; an unreachable cecum or ileum; known preexisting polyps; and inadequate and poor bowel preparation evaluated by the Aronchick scale. Propensity score matching (PSM) was performed to balance baseline characteristics between groups. Variables used in the matching were age, sex, number of examinations, reason for examination, bowel preparation, and endoscopist experience.

### Endoscopic procedures

Hamamatsu University Hospital is a tertiary care center that performs colonoscopy using two types of video processor systems: the LUCERA and the X1 systems. The new X1 system features novel five-LED illumination technology and incorporates a CMOS image sensor distinct from the conventional CCD sensor.

Patients were informed by outpatient physicians regarding indications for colonoscopy, procedure details, potential risks, and personal data protection measures, and written informed consent was obtained. Patients were able to schedule the time and date of their colonoscopy; however, they could not choose the endoscopy system used.

Bowel preparation consisted of 1 to 2 L of a polyethylene glycol solution containing ascorbic acid, followed by an additional 1 to 2 L of water. Sedated colonoscopy was performed at patient request, with midazolam (2–5 mg) and/or pentazocine (7.5–15 mg) administered as sedatives.

In this study, IEE techniques—such as chromoendoscopy, TXI, and NBI—were not routinely used for polyp detection but were applied at the discretion of the endoscopist for detailed examination. When endoscopists detected lesions suspected to be adenomas (excluding clearly benign lesions such as rectal hyperplastic lesions), they opted for endoscopic resection and submitted the specimens for pathological diagnosis.

Withdrawal time of the colonoscopy was calculated based on endoscopist-reported data in the Solemio QUEV system. Because both insertion time and total procedure time were recorded in the system, withdrawal time was calculated as (total procedure time - insertion time).

Quality of bowel preparation was assessed based on the Aronchick scale and categorized into five levels: excellent, good, fair, poor, and inadequate. Patients with poor and inadequate preparation were excluded from the analysis.

### Endoscopists

A total of 54 endoscopists were involved in the study, of whom 45 were classified as non-trainees and nine as trainees. A trainee was defined as a doctor who has performed less than 300 colonoscopies.

### Endpoints

The primary endpoint was the comparison of the ADR between the two groups using different video processor systems. ADR was defined as the proportion of patients with at least one adenoma detected among all patients examined. Secondary endpoints included PDR, advanced ADR (AADR), sessile serrated lesion detection rate (SSLDR), number of adenomas per colonoscopy (APC), and number of polyps per colonoscopy (PPC), including both adenomas and hyperplastic polyps. An advanced adenoma was defined as an adenoma measuring ≥ 10 mm, exhibiting (tubulo)villous histology, or showing high-grade dysplasia. APC, PPC, and SSL per colonoscopy (SSLPC) were defined as the mean number of adenomas, polyps, and SSLs detected per colonoscopy, respectively.

### Characteristics and classification of polyps

In this study, only polyps that were detected by endoscopy, resected during the same procedure, and confirmed by pathological diagnosis were included in the analysis. According to the guidelines of the Japanese Society of Gastroenterology 14 , hyperplastic polyps of 5 mm or less in the rectum and sigmoid colon diagnosed by endoscopists were not resected as a general rule, and therefore, these lesions were excluded from the analysis. Data regarding location, size, macroscopic morphology, and pathological diagnosis of the polyps were collected. Polyp morphology was classified according to the Paris classification.

### Statistical analysis

Statistical analyses were performed using SPSS version 27 (IBM, Armonk, New York, United States) and the EZR plugin for R (Saitama Medical Center, Jichi Medical University, Saitama, Japan), a modified version of R Commander designed to add statistical functions commonly used in biostatistics. Continuous data are presented as mean ± standard deviation and were analyzed using the Student’s *t* -test or the Mann-Whitney U test, depending on the data distribution. Categorical data were analyzed using Pearson’s chi-square test or Fisher’s exact test, as appropriate. Statistical significance was defined as *P* < 0.05. Multivariate analysis was performed using a logistic regression model to assess the association between clinical factors and adenoma detection. Variables found to be significantly associated in the univariate analysis were included as covariates in the multivariate model.

## Results

### Patient enrollment and baseline characteristics

**Fig. 2:**
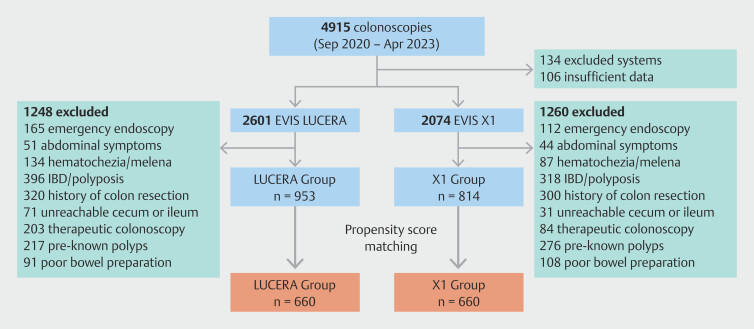
Flow chart depicting patient enrollment.

Fig. 2 shows the flowchart of patient enrollment. A total of 4,915 eligible patients were assessed for trial participation during the study period and 106 patients were excluded due to insufficient medical records. Of them, 2,601 underwent colonoscopy using the LUCERA system and 2,074 using the X1 system. After further exclusion based on predefined criteria, 953 cases were included in the LUCERA group and 814 in the X1 group.

Demographic and clinical characteristics of patients were compared between the two groups. There were no significant differences in age, gender, bowel preparation quality, number of previous colonoscopies, or endoscopist experience. However, significant differences were observed in the reason for examination, intubation time, and withdrawal time, which could potentially affect the results ( **Supplementary Table 1** ).

PSM was performed to balance baseline characteristics. After matching, 660 cases were included in each group and all characteristics were well balanced ( Table 1 ). These matched cases were used for subsequent analysis.

**Table:** **Table 1** Demographic and colonoscopy characteristics of propensity score matched cohort.

**Group**	**EVIS LUCERA**	**EVIS-X1**	***P* value **
Numbers of patients	660	660	
Gender; male/female, n (%)	424 (64.2)/236 (35.8)	407 (61.7)/253 (38.3)	0.362
Age, mean ± SD (range), years	66.22 ± 14.07	66.16 ± 14.68	0.939
Number of examinations, n (%)			0.654
First time	239 (36.2)	247 (37.4)	
Second time or more	372 (56.4)	372 (56.4)	
unknown	49 (7.4)	41 (6.2)	
Reason for examination, n (%)			0.958
Screening	438 (66.4)	441 (66.8)	
Surveillance	119 (18.0)	120 (18.2)	
FIT positive	103 (15.6)	99 (15.0)	
Bowel preparation, n (%)			0.63
Excellent	199 (30.2)	210 (31.8)	
Good	321 (48.6)	323 (48.9)	
Fair	140 (21.2)	127 (19.2)	
Endoscopist experience, n (%)			1
Non-trainee (≥ 300 cases)	519 (78.6)	519 (78.6)	
Trainee (< 300 cases)	141 (21.4)	141 (21.4)	
Withdrawal time, minutes ± SD	14.52 ± 11.95	15.35 ± 11.04	0.257
Intubation time, minutes ± SD	11.14 ± 8.34	11.70 ± 9.08	0.219
FIT, fecal immunochemical test; SD, standard deviation.			

### Comparison of adenoma and polyp detection between LUCERA and X1 groups

As summarized in Table 2, ADR, and PDR were slightly higher in the X1 group compared with the LUCERA group: 36.1% vs. 32.1% ( *P* = 0.147), and 36.8% vs. 40.9% ( *P* = 0.142), respectively, although these differences were not statistically significant.

**Fig. 3:**
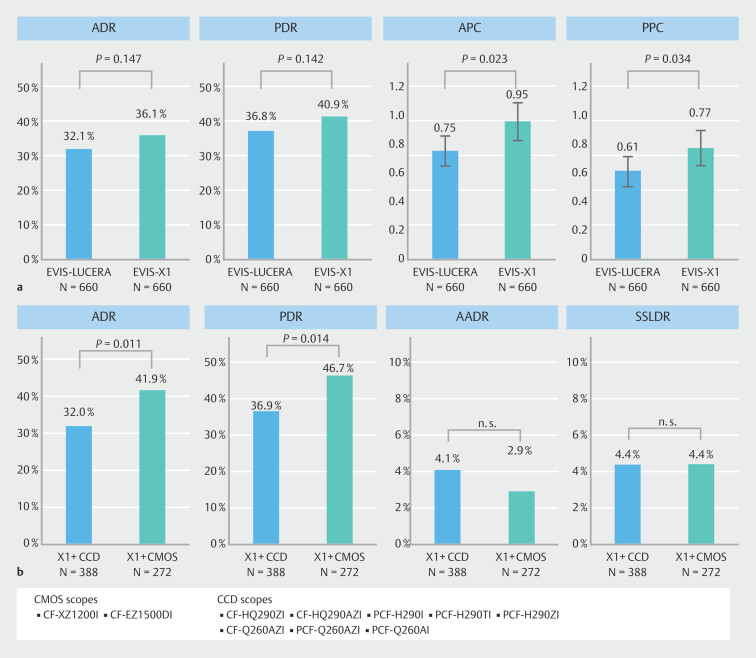
Comparison of adenoma and polyp detection. **a** Comparison of ADR, PDR, APC, and PPC between X1 group and LUCERA group. **b** Comparison of ADR, PDR, AADR, and SSLDR between CMOS and CCD scopes in the X1 group.

APC was significantly higher in the X1 group compared with the LUCERA group (0.77 vs. 0.61, *P* = 0.034), as was the PPC (0.95 vs. 0.75, *P* = 0.023) ( Fig. 3 **a** ).

AADR, SSLDR, AAPC, and SSLPC did not show statistically significant differences between the X1 and LUCERA groups ( Table 2 ).

**Table:** **Table 2** Outcome comparison between EVIS-X1 and EVIS LUCERA groups.

**Group**	**EVIS LUCERA**	**EVIS-X1**	***P* value **
Numbers of patients	660	660	
ADR, %	32.1	36.1	0.147
PDR, %	36.8	40.9	0.142
AADR, %	5.6	3.6	0.115
SSLDR, %	3.0	4.4	0.244
APC, mean ± SD	0.61 ± 1.25	0.77 ± 1.54	0.034
PPC, mean ± SD	0.75 ± 1.44	0.95 ± 1.74	0.023
AAPC, mean ± SD	0.06 ± 0.28	0.05 ± 0.28	0.380
SSLPC, mean ± SD	0.05 ± 0.40	0.06 ± 0.33	0.454
AADR, advanced adenoma detection rate; AAPC, advanced adenomas per colonoscopy; ADR, adenoma detection rate; APC, adenomas per colonoscopy PDR, polyp detection rate; PPC, polyps per colonoscopy; SSLDR, sessile serrated lesion detection rate; SSLPC, sessile serrated lesion per colonoscopy.			

### Comparison of adenoma and polyp detection between CMOS and CCD scopes in the X1 group

As part of a subgroup analysis, we further evaluated the effects of the new colonoscopes equipped with CMOS sensors. CMOS is a high-performance image sensor commonly used in devices such as single-lens reflex cameras, and enables brighter, lower-noise, higher-quality imaging.

As shown in Fig. 3 **b** , scopes equipped with CMOS image sensors (CF-XZ1200I, CF-EZ1500DI) demonstrated a significantly higher ADR (41.9%) compared with scopes equipped with traditional CCD sensors within the X1 group. However, when CCD scopes were used with the X1 system, the ADR did not improve compared with the LUCERA system. We hypothesize that use of CCD scopes may act as a bottleneck, preventing the system from achieving its optimal performance.

### Comparison of characteristics of detected polyps between LUCERA and X1 groups.

We evaluated characteristics of the detected polyps. A total of 1,124 polyps were endoscopically resected and analyzed, including 910 (81.0%) adenomas, 70 (6.2%) SSLs, and 67 (6.0%) hyperplastic polyps ( **Supplementary Table 2** ). As shown in Table 3 , there were no significant differences in location, size, or morphology of polyps between the LUCERA and X1 groups. As an additional analysis, we examined differences between CMOS and CCD scopes within the X1 group. In the subgroup analysis, mean polyp size was significantly smaller in the CMOS group; however, distribution across the three size categories (< 5 mm, 5–9 mm, and ≥ 10 mm) did not differ significantly between groups. No significant differences were observed in terms of morphology and location ( Table 4 ).

**Table:** **Table 3** Polyp detection according to size, morphology, and site in the EVIS-X1 and EVIS LUCERA groups.

**Group**	**EVIS LUCERA**	**EVIS-X1**	***P* value **
Numbers of polyps	497	627	
Polyp size, mm ± SD	6.02 ± 4.99	6.11 ± 4.33	0.753
Polyp size (%)			0.182
Small (< 5 mm)	210 (42.3)	231 (36.8)	
Medium (5 mm - 9 mm)	236 (47.5)	326 (52.0)	
Large (≥ 10 mm)	51 (10.3)	70 (11.2)	
Morphology			
0-Ip	29 (5.8)	28 (4.5)	
0-Isp	109 (21.9)	137 (21.9)	
0-Is	280 (56.3)	345 (55.0)	
0-IIa	79 (15.9)	116 (18.5)	
0-IIb	0	1 (0.2)	
0-IIc	0	0	
Morphology (subclassification)			0.329
Pedunculated	138 (27.8)	165 (26.3)	
Sessile	280 (56.3)	345 (55.0)	
Flat	79 (15.9)	117 (18.7)	
Site			
Cecum	28 (5.6)	44 (7.0)	
Ascending colon	107 (21.5)	133 (21.2)	
Transverse colon	114 (22.5)	155 (24.7)	
Descending colon	68 (13.7)	68 (10.8)	
Sigmoid colon	143 (28.8)	172 (27.4)	
Rectum	39 (7.8)	55 (8.8)	
Site (subclassification)			0.306
Right sided	247 (49.7)	332 (53.0)	
Left sided	250 (50.3)	295 (47.0)	
SD, standard deviation.			

**Table:** **Table 4** Polyp detection according to size, morphology and site in the EVIS-X1 with CMOS and the other CCD systems.

**Group**	**CCD group**	**CMOS group**	***P* value **
Number of polyps	371	256	
Polyp size, mm ± SD	6.11 ± 4.08	5.23 ± 2.45	0.002
Polyp size (%)			0.066
Small (< 5 mm)	129 (34.8)	102 (39.8)	
Medium (5 mm - 9 mm)	192 (51.8)	134 (52.3)	
Large (≥ 10 mm)	50 (13.5)	20 (7.8)	
Morphology			
0-Ip	19 (5.1)	9 (3.5)	
0-Isp	76 (20.5)	61 (23.8)	
0-Is	206 (55.5)	139 (54.3)	
0-IIa	69 (18.6)	47 (18.4)	
0-IIb	1 (0.3)	0	
0-IIc	0	0	
Morphology (subclassification)			0.612
Pedunculated	19 (5.1)	9 (3.5)	
Sessile	282 (76.0)	200 (78.1)	
Flat	70 (18.9)	47 (18.4)	
Site			
Cecum	30 (8.1)	14 (5.5)	
Ascending colon	61 (16.4)	72 (28.1)	
Transverse colon	106 (28.6)	49 (19.1)	
Descending colon	40 (10.8)	28 (10.9)	
Sigmoid colon	101 (27.2)	71 (27.7)	
Rectum	33 (8.9)	22 (8.6)	
Site (subclassification)			0.933
Right sided	197 (53.1)	135 (52.7)	
Left sided	174 (46.9)	121 (47.3)	
CCD, charge coupled device; CMOS, complementary metal oxide semiconductor; SD, standard deviation.			

### Univariate and multivariate analysis of factors associated with adenoma detection

Finally, logistic regression analysis was performed to identify factors associated with ADR in this study. Variables examined included age, sex, use of the X1 system, use of the X1 system with a CMOS scope, positive fecal occult blood test (FOBT), endoscopist experience, bowel preparation quality, first-time examination, and right-sided colon involvement. As shown in Table 5 , univariate analysis identified age, sex, use of the X1 system with a CMOS scope, positive FOBT, and first-time examination as significantly associated factors. Multivariate analysis of these five factors revealed that age, sex, use of the X1 system with a CMOS scope, and positive FOBT were independent factors associated with adenoma detection.

**Table:** **Table 5** Univariate and multivariate analysis for factors related to adenoma detection.

	**Univariate analysis**			**Multivariate analysis**		
**Factor**	**Crude OR**	**95% CI**	***P* value **	**Adjusted OR**	**95% CI**	***P* value **
Age, over 60	2.300	1.800–2.950	< 0.0001	2.190	1.690–2.840	< 0.0001
Sex, Male	1.610	1.320–1.980	< 0.0001	1.500	1.200–1.860	0.0003
System, EVIS-X1	1.210	0.994–1.470	0.0569			
System, EVIS-X1 with CMOS scope	1.470	1.150–1.880	0.0019	1.520	1.170–1.980	0.0018
Reason, FIT positive	1.840	1.430–2.370	< 0.0001	2.020	1.540–2.650	< 0.0001
Endoscopist experience, non-trainee	0.860	0.688-1.080	0.1880			
Bowel preparation, excellent	0.943	0.761–1.170	0.5941			
Number of examinations,first time	0.749	0.606-0.925	0.0073	0.813	0.652-1.010	0.0646
Colon side, right	1.003	0.863-1.166	0.9694			
FIT, fecal immunochemical test; OR, odds ratio.						

## Discussion

This study is the first to report on the impact of a colonoscopy system featuring both five-LED illumination and a novel CMOS image sensor on colorectal adenoma detection. Using PSM to balance baseline characteristics, we compared the EVIS X1 system with the EVIS LUCERA ELITE system. Although no statistically significant difference in ADR was observed between the two groups, the X1 group demonstrated significantly higher APC and PPC, suggesting a potential benefit in detecting additional lesions. Furthermore, within the X1 group, scopes equipped with CMOS image sensors achieved notably higher ADR compared with those with conventional CCD sensors. These findings suggest that the X1 system, particularly when combined with CMOS-equipped scopes, has the potential to enhance the quality of endoscopic screening for colorectal neoplasms.

Many studies to date have investigated usefulness of IEE techniques, such as chromoendoscopy 15 , TXI 16 , LCI 17 , and NBI 6 , for detection of colorectal adenomas. However, use of these techniques often requires extended examination time due to the need for additional observations, making their consistent application in universal screening settings impractical. Regarding xenon light endoscopic systems, several studies have compared high-resolution colonoscopes with earlier generations of standard-resolution scopes, reporting that high-resolution colonoscopy improves ADR 11 18 19 . A meta-analysis of five studies involving 4,422 average-risk patients demonstrated a 3.5% incremental yield (95% confidence interval [CI], 0.9%-6.1%) for HD-WLE compared with SD-WLE in detecting patients with at least one adenoma 12 . In addition, a more recent meta-analysis of six randomized controlled trials (RCTs) involving 4,594 patients found that ADR was significantly higher with HD-WLE than with SD colonoscopy (40% vs. 35%; relative risk [RR], 1.13; 95% CI, 1.05–1.22; *P* = 0.001) 20 . Olympus H290 LUCERA ELITE HD colonoscopes have also been shown to improve adenoma detection in moderate-risk populations, with an estimated 12% improvement in ADR potentially translating into a significant reduction in colorectal cancer mortality 21 . Based on these findings, we hypothesized that the X1 system, incorporating five-LED illumination technology and a new CMOS image sensor, would further increase the ADR by providing higher image resolution than ever before.

First, we compared detection rates for polyps of any size, including ADR, APC, and PPC, between the X1 and LUCERA groups. Our results showed that both APC and PPC were significantly higher in the X1 group than in the LUCERA group. These findings suggest that the X1 system may substantially reduce the miss rate for colorectal lesions. Polyp miss rates during colonoscopy have been reported to be influenced by colonoscopist experience 22 23 . To investigate this, we evaluated polyp detection success among trainees, defined as endoscopists with experience with fewer than 300 cases, and non-trainees, defined as those with experience with 300 cases or more. We found no significant difference in detection rates between trainees and non-trainees ( **Supplementary Table 3** ). At our hospital, trainees never perform colonoscopy independently but always conduct examinations under the supervision of non-trainee endoscopists. Thus, we hypothesize that the skills of non-trainee endoscopists may have contributed to the high detection rates observed among trainees. These results suggest that the X1 system may serve as a valuable diagnostic tool for colonoscopists regardless of their level of experience.

A previous report has shown that adenoma detection using HD-WLE with xenon light sources was significantly higher compared with SD-WLE for flat adenomas (9.5% vs. 2.4%, *P* = 0.003) and right-sided adenomas (34.0% vs. 19.0%, *P* = 0.001) in a two-center RCT 24 . Detection of diminutive polyps (< 5 mm) was also significantly increased with HD-WLE (22.5% vs. 15.6%, *P* < 0.001), as was the adenoma-per-patient rate (0.57 vs. 0.47, *P* < 0.001) 25 . In our study, LED-based endoscopes showed no overall advantage for right-sided or small lesions. Interestingly, subgroup analysis indicated a modest benefit for small-lesion detection with CMOS scopes, but this was not significant when stratified by three lesion sizes. Further, AADR in the X1 group was lower than in the LUCERA group (3.6% vs. 5.6%, *P* = 0.115). These findings suggest that although LED systems may aid small-lesion detection, their impact on colorectal cancer prevention via increased polyp detection rates is limited and may not translate into higher AADR. In recent years, usefulness of artificial intelligence (AI)-assisted diagnostic support for colon polyp detection has been increasingly reported 26 27 . AI diagnostic support is expected to complement or even replace traditional IEE and may become the next major modality integrated into high-resolution endoscopes with LED light sources for screening colonoscopy.

There were several limitations in this study. First, it was a single-center study, and endoscopists were not blinded to group assignment. Second, this study had inherent limitations associated with retrospective data collection, and some potential confounding factors—such as body mass index, smoking status, alcohol intake, and medication use—may not have been fully accounted for in the analysis. Third, biopsy time and polyp removal time were not precisely recorded and subtracted, potentially leading to overestimation of withdrawal time. Although we attempted to balance withdrawal times using PSM, withdrawal time excluding treatment procedures may have been shorter in the X1 group. Therefore, a larger RCT with precise adjustment for withdrawal time is advisable. Fourth, although scope allocation was generally decided by the technicians based on post-cleaning availability, we cannot completely exclude the possibility of introducing selection bias into subgroup analyses. Furthermore, post-hoc power analysis indicated that the study had approximately 33.5% power to detect a 4% difference in ADR, suggesting a potential risk of type II error. Future larger-scale prospective studies are needed to confirm the benefit of the EVIS X1 system.

## Conclusions

Despite these limitations, our findings suggest that the X1 system, particularly when combined with CMOS image sensor-equipped endoscopes, has the potential to improve detection of colorectal adenomas and polyps, thereby enhancing the quality of colonoscopy screening for colorectal cancer. We hope that the results of this study will contribute to development of effective colonoscopic screening strategies in the future.

## Supplementary Material

Supplementary Material Zusatzmaterial
