# Supplementary material for: Comparison of adenoma detection rate using the novel 5-LED vs xenon-light endoscopic system: Propensity score matching analysis
Source: Endosc Int Open. 2025 Dec 19;13:a27606529. doi: 10.1055/a-2760-6529 (PMC12818183; doi:10.1055/a-2760-6529)
Supplement: Supplementary file 1 — Supplementary Material [file 10-1055-a-2760-6529_27654789.pdf]

Supplementary Table 1 Demographic and colonoscopy characteristics of all study participants.

Group	EVIS-LUCERA	EVIS-X1	*P* value
Numbers of patients	953	814	
Gender; male/female, n (%)	291 (56.0)/229 (44.0)	309 (59.4)/211 (40.6)	0.435
Age, mean ± SD (range), years	65.78 ± 14.66	66.63 ± 14.20	0.217
Number of examinations, n (%)			0.703
First time	341 (35.8)	286 (35.1)	
Second time or more	548 (57.5)	465 (57.1)	
unknown	64 (6.7)	63 (7.7)	
Reason for examination, n (%)			0.024
Screening	579 (60.8)	545 (67.0)	
Surveillance	199 (20.9)	147 (18.1)	
FIT positive	175 (18.4)	122 (15.0)	
Bowel preparation, n (%)			0.506
Excellent	286 (30.0)	231 (28.4)	
Good	491 (51.5)	416 (51.1)	
Fair	176 (18.5)	167 (20.5)	
Endoscopist experience, n (%)			< 0.001
Non-trainee (≥ 300 cases)	810 (85.0)	520 (63.9)	
Trainee (< 300 cases)	143 (15.0)	294 (36.1)	
Withdrawal time, minutes ± SD	14.31 ± 12.68	16.78 ± 11.76	< 0.001
Intubation time, minutes ± SD	10.57 ± 8.17	12.56 ± 9.95	< 0.001

FIT, fecal immunochemical test; SD, standard deviation.

Supplementary Table 2 Characteristics of detected lesions.

Characteristics	All polyps (N = 1124)
Polyp size, mm ± SD	5.64 ± 3.49
Small (< 5 mm)	441 (39.2)
Medium (5 mm-9 mm)	562 (50.0)
Large (≥ 10 mm)	121 (10.8)
Site	
Cecum	72 (6.4)
Ascending colon	240 (21.4)
Transverse colon	267 (23.8)
Descending colon	136 (12.1)
Sigmoid colon	315 (28.0)
Rectum	94 (8.4)
Right sided	579 (51.5)
Left sided	545 (48.5)
Morphology	
0-Ip	57 (5.1)
0-Isp	246 (21.9)
0-Is	625 (55.6)
0-IIa	195 (17.3)
0-IIb	1 (0.1)
0-IIc	0
Pedunculated	303 (27.0)
Sessile	625 (55.6)
Flat	196 (17.4)

Pathological classification	
Adenoma	910 (81.0)
Severe dysplasia	75 (6.7)
Mild-moderate dysplasia	835 (74.3)
Sessile serrated lesion	70 (6.2)
Hyperplastic polyp	67 (6.0)
Inflammatory polyp	9 (0.8)
Carcinoma	31 (2.8)
Normal mucosa	21 (1.9)
Others*	5 (0.4)
Unknown	10 (0.9)

*Granuloma, juvenile polyp, leiomyoma, mesenchymal polyp P-J polyp, Schwann cell hamartoma.

SD, standard deviation.

Supplementary Table 3 Polyp and adenoma detection in EVIS-X1 and EVIS-LUCERA groups in non-trainees and trainees.

**Group**	**Trainee (< 300 cases)**	**Non-trainee (≥ 300 cases)**	***P* value**
Numbers of patients	282	1038	
ADR, %	34.0	34.1	1
PDR, %	33.7	33.0	0.831
AADR, %	5.7	4.3	0.339
SSLDR, %	3.9	3.7	0.859
APC, mean ± SD	0.70 ± 1.35	0.69 ± 1.42	0.880
PPC, mean ± SD	0.85 ± 1.46	0.85 ± 1.63	0.981
AAPC, mean ± SD	0.07 ± 0.29	0.05 ± 0.28	0.478
SSLPC, mean ± SD	0.04 ± 0.19	0.06 ± 0.40	0.470
Withdrawal time, minutes ± SD	18.75 ± 12.51	14.32 ± 10.88	< 0.001
Intubation time, minutes ± SD	17.33 ± 8.67	10.22 ± 8.24	< 0.001

ADR, adenoma detection rate; AADR, advanced adenoma detection rate; AAPC, advanced adenomas per colonoscopy; APC, adenomas per colonoscopy; PDR, polyp detection rate; PPC, polyps per colonoscopy; SSLDR, sessile serrated lesion detection rate; SSLPC, sessile serrated lesion per colonoscopy.
